# Microbial epidemiology and risk factors for relapse in gram-negative bacteria catheter-related bloodstream infection with a pilot prospective study in patients with catheter removal receiving short-duration of antibiotic therapy

**DOI:** 10.1186/s12879-020-05312-z

**Published:** 2020-08-17

**Authors:** Bhitta Surapat, Preecha Montakantikul, Kumthorn Malathum, Sasisopin Kiertiburanakul, Pitak Santanirand, Busba Chindavijak

**Affiliations:** 1 Department of Pharmacy, Faculty of Medicine Ramathibodi Hospital, Mahidol University, Bangkok, Thailand; 2 Department of Pharmacy, Faculty of Pharmacy, Mahidol University, Bangkok, Thailand; 3 Department of Medicine, Faculty of Medicine Ramathibodi Hospital, Mahidol University, Bangkok, Thailand; 4 Department of Pathology, Faculty of Medicine Ramathibodi Hospital, Mahidol University, Bangkok, Thailand

**Keywords:** Duration, Catheter removal, *Pseudomonas aeruginosa*

## Abstract

**Background:**

Infectious Diseases Society of America (IDSA) guidelines suggest 7–14 days’ duration of antibiotic treatment for uncomplicated Gram-negative bacteria (GNB) catheter-related bloodstream infection (CRBSI). The objectives of this study were to review microbial epidemiology, to determine rate and risk factors for relapse, and to compare clinical outcomes in patients receiving long- versus short-duration antibiotic therapy.

**Methods:**

A retrospective phase 1 study was conducted between January 2010 and October 2016 to review microbial epidemiology and to determine the incidence of and risk factors for relapse in patients with GNB CRBSI, according to the IDSA guidelines diagnostic criteria. In phase 2 of the study, patients without risk factors for relapse between November 2016 and October 2017 were prospectively recruited to receive antibiotic therapy for 7 days after catheter removal. Matched patients from the retrospective phase 1 study who had received antibiotic therapy for ≥14 days were selected as a phase 2 control group to compare outcomes.

**Results:**

In phase 1, three most common pathogens identified among 174 cases were *Pseudomonas aeruginosa* (22.0%)*, Klebsiella pneumoniae* (16.7%), *and Stenotrophomonas maltophilia* (13.4%)*.* Eighty-nine episodes of infection occurred while patients were receiving antibiotic therapy. Of 140 cases, the relapse rate was 6.4%. Catheter retention was the only risk factor strongly associated with relapse (odds ratio = 145.32; 95% confidence interval 12.66–1667.37, *P* < 0.001). In phase 2, 11 patients with catheter removal were prospectively recruited to receive short-duration therapy. The number of patients with relapse receiving long- or short-duration therapy was 1 (3%) and 0 (0%), respectively (*P* = 1.000).

**Conclusions:**

For the management of patients with uncomplicated GNB CRBSI, empiric broad-spectrum antibiotic therapy with adequate coverage of *P. aeruginosa* should be chosen. Catheter removal should be performed to prevent relapse and shortening the duration of treatment could be considered.

**Trial registration:**

Thai Clinical Trial Registry: TCTR20190914001. Retrospectively registered on 13 September 2019.

## Background

Catheter-related bloodstream infection (CRBSI) is one of the most important healthcare-associated infections. In the past, the most common causative pathogens of CRBSI were Gram-positive bacteria, such as coagulase-negative staphylococci and *Staphylococcus aureus* [1–3]. However, recent global data have reported that the epidemiology of CRBSI has changed, with a predominance of Gram-negative bacteria (GNB), such as *Pseudomonas aeruginosa*, *Klebsiella* spp., or *Acinetobacter* spp. as the causative pathogen [4–7].

For the treatment of uncomplicated GNB CRBSI, the clinical practice guidelines for the diagnosis and management of intravascular catheter-related infection by the Infectious Diseases Society of America (IDSA) suggests antibiotic therapy of 7–14 days [8]. However, this treatment recommendation came from expert opinions, which is different from recommendations for Gram-positive CRBSI that were based on stronger and better quality of evidence [8]. In Thailand, there is no local treatment guidelines and the IDSA guidelines are mainly followed by Thai physicians. Despite the 7–14 day range for treatment suggested in the guidelines [8], most physicians prefer to prolong antibiotic therapy to 10–14 days, even in patients whose catheters have been removed. Understanding the nature of this disease in terms of relapse could help reducing unnecessary antibiotic use. Thus, the objectives of this study were to review microbial epidemiology to determine relapse rates, and to identify patients with low risk for relapse, as potential candidates for short-duration antibiotic therapy.

## Methods

### Setting and patients

The study was conducted in two phases at Ramathibodi hospital, Mahidol University (a 1200-bed university hospital in Bangkok, Thailand). Phase 1 was a retrospective cohort study to review microbial epidemiology and to find the incidence and risk factors for relapse in patients with GNB CRBSI. Phase 2 was a nonrandomized, historically controlled study to compare rates of relapse in GNB CRBSI patients with catheter removal with low risk of relapse who received long- versus short-duration antibiotic treatment.

Phase 1 collected data from hospitalized adult patients aged ≥18 years between January 2010 and October 2016. These patients had culture proven GNB CRBSI, according to 2009 IDSA diagnostic criteria (patients with bacteremia with differential time to catheter culture positivity versus peripheral hemoculture of > 2 h, or semiquantitative catheter culture of > 15 colony-forming unit per catheter segment whereby the same organism is recovered from the catheter segment and the peripheral hemoculture) [8]. This included patients with nosocomial infection, which was defined as patients with positive hemoculture obtained > 48 h after hospital admission or within 48 h after discharge [9]. Patients who had positive hemoculture obtained within 48 h of admission were classified as community-acquired infection, except for those who attended hemodialysis clinic or received intravenous chemotherapy within 30 days, or were hospitalized for > 2 days within 90 days would be classified as healthcare-associated infection [10]. In the clinical analysis, all included patients must have been administered appropriate antibiotic treatment. Appropriate treatment was defined as receiving at least one active agent based on an in vitro susceptibility profile via a systemic route (intravenous or oral) with or without antibiotic lock therapy. Patients with persistent bacteremia, suppurative thrombophlebitis, septic emboli, or other metastatic infectious complications, such as infective endocarditis, septic arthritis, osteomyelitis, spondylodiscitis, and deep-seated abscess were excluded.

Phase 2 recruited hospitalized adult patients aged ≥18 years, between November 2016 and October 2017. These patients had GNB CRBSI, according to the 2009 IDSA definition, and were treated with appropriate antibiotics. Patients were excluded if they (1) had not had their catheters removed; (2) had risk factors for relapse according to the phase 1 study’s results; (3) had a prosthetic valve or synthetic endovascular grafts; (4) had neutropenia (absolute neutrophil count < 500/mm^3^), as evaluated on their antibiotic discontinuation day; (5) had persistent bacteremia, suppurative thrombophlebitis, septic emboli, or other metastatic infectious complications, such as infective endocarditis, septic arthritis, osteomyelitis, spondylodiscitis, or deep-seated abscess, as evaluated on their antibiotic discontinuation day; or (6) had other indications for receiving antibiotics with the activity against the causative CRBSI pathogen for more than 7 days.

### Data collection

Patients’ demographic and clinical data were recorded. Patients were monitored for outcomes (relapse and death) in a follow-up period of 30 days after antibiotic discontinuation. Because some patients might receive an antibiotic that was active against the CRBSI pathogen during the follow-up period for other reasons, the time to next antibiotic use and its indication were also recorded.

### Intervention and data analysis

In the retrospective phase 1 study, microbiology data from all cases with GNB CRBSI were reviewed, but only cases that met the inclusion and exclusion criteria were selected for clinical analysis. The distribution of antibiotic duration was analyzed and a cut-off point was determined to divide patients into two groups; the long-duration group and the short-duration group. Patients’ baseline characteristics and clinical outcomes were compared between the two groups. For the categorical variables, the chi-squared test was used. For the continuous variables, the Shapiro–Wilk test was used to test for normality. The Mann–Whitney test and an unpaired t test were used for the comparison if the data were non-normally distributed and normally distributed, respectively. Variables with significances of *p* < 0.05 in the bivariate analysis were included in the multiple logistic regression analysis to identify factors associated with relapse or mortality, and to determine whether shortening the duration of antibiotic therapy was associated with poor outcomes.

For the prospective phase 2 study, patients were assigned to receive short-duration antibiotic therapy for 7 days. The choice of antibiotic was made by their physicians. The duration of therapy was counted from the first day that patients had their catheters removed and received appropriate antibiotic treatment until that treatment was interrupted. Total duration of antibiotics including treatment before catheter removal were no longer than 10 days. Catheter reinsertion could be performed only after the patients had received appropriate antibiotic treatment. Two specimens of hemoculture were taken at 7 days after discontinuation of antibiotic therapy, as a patient safety surveillance measure. Cases with positive surveillance hemoculture were treated by physicians according to standards of care.

Patients without risk factor for relapse found in the phase 1 study, who received antibiotic therapy of at least 14 days, were selected from the phase 1 study to form a long-duration group for phase 2. The ratio between the long- and short-duration groups was 3:1. Patients were selected consecutively with best-matched characteristics, consisting of immunocompromised status, age, underlying cancer, ICU admission at onset of CRBSI, and Charlson comorbidity index. These characteristics were chosen because of their possible impact on the patients’ immune response and clinical outcomes. Baseline characteristics and clinical outcomes were then compared between the two groups, using statistics, as in the phase 1 study. All statistical analyses were performed using IBM® SPSS® Statistics version 18.

## Results

A total of 174 cases with a confirmed diagnosis of GNB CRBSI were identified. Microbial etiology was monomicrobial in 161 cases and polymicrobial in 13 cases (2 cases of 1 GNB and 1 Gram-positive bacteria, 10 cases of 2 GNB, and 1 case of 3 GNB). Various GNB pathogens were found, as shown in Table 1. Three most common pathogens associated with nosocomial infections were *P. aeruginosa* (23.2%), *Klebsiella pneumoniae* (17.2%), and *Acinetobacter baumannii* (15.2%). For healthcare-associated infections, four common pathogens were *P. aeruginosa* (17.6%), *Burkholderia cepacia* complex (17.6%), *K. pneumoniae* (14.7%), and *Enterobacter cloacae* (14.7%)*.*

**Table 1:** Gram-negative bacteria associated with catheter-related bloodstream infection according to origin of infection

Organisms	Total (%) (*N* = 186)	Origin of infection		
		Community-acquired (%) (*N* = 1)	Healthcare-associated (%) (*N* = 34)	Nosocomial (%) (*N* = 151)
*Pseudomonas aeruginosa*	41 (22.0)	–	6 (17.6)	35 (23.2)
*Klebsiella pneumoniae*	31 (16.7)	–	5 (14.7)	26 (17.2)
*Stenotrophomonas maltophilia*	25 (13.4)	–	3 (8.8)	22 (14.6)
*Acinetobacter baumannii*	24 (12.9)	–	1 (2.9)	23 (15.2)
*Escherichia coli*	18 (9.7)	–	1 (2.9)	17 (11.3)
*Burkholderia cepacia* complex	12 (6.5)	–	6 (17.6)	6 (4.0)
*Enterobacter cloacae*	11 (5.9)	–	5 (14.7)	6 (4.0)
*Serratia marcescens*	6 (3.2)	–	–	6 (4.0)
*Enterobacter aerogenes*	4 (2.2)	–	2 (5.9)	2 (1.3)
*Chryseobacterium indologenes*	2 (1.1)	–	–	2 (1.3)
*Elizabethkingia meningoseptica*	2 (1.1)	–	–	2 (1.3)
*Ralstonia (Pseudomonas) pickettii*	2 (1.1)	–	2 (5.9)	–
*Acinetobacter baylyi*	1 (0.5)	–	–	1 (0.7)
*Acinetobacter junii*	1 (0.5)	1 (100.0)	–	–
*Acinetobacter nosocomialis*	1 (0.5)	–	1 (2.9)	–
*Aeromonas hydrophila*	1 (0.5)	–	1 (2.9)	–
*Citrobacter freundii*	1 (0.5)	–	–	1 (0.7)
*Ochrobactrum anthropi*	1 (0.5)	–	1 (2.9)	–
*Proteus mirabilis*	1 (0.5)	–	–	1 (0.7)
*Pseudomonas chlororaphis*	1 (0.5)	–	–	1 (0.7)

Of all, 89 episodes of infection occurred while patients were receiving antibiotic therapy. The most common receiving antibiotics were carbapenems, piperacillin/tazobactam, and vancomycin. Breakthrough infections were likely caused by *Acinetobacter* spp., *Pseudomonas* spp., and *Stenotrophomonas maltophilia.* Organisms associated with breakthrough infections among patients receiving each antibiotic are shown in Table 2.

**Table 2:** Antibiotic-breakthrough catheter-related bloodstream infection pathogens according to receiving antibiotics

Receiving antibiotics	Total	Organisms												
		*Acinetobacter* spp*.*	*Pseudomonas* spp*.*	*Stenotrophomonas maltophilia*	*Klebsiella pneumoniae*	*Burkholderia cepacia* complex	*Escherichia coli*	*Enterobacter cloacae*	*Serratia marcescens*	*Elizabethkingia meningoseptica*	*Citrobacter freundii*	*Proteus mirabilis*	*Chryseobacterium indologenes*	*Ralstonia pickettii*
Carbapenems^a^	23	9	6	4	2	1	–	–	–	–	–	–	1	–
Piperacillin/tazobactam	15	2	4	1	1	3	1	1	1	–	1	–	–	–
Vancomycin	14	5	2	3	2	–	1	–	–	–	–	–	–	1
Metronidazole	12	6	3	1	–	–	2	–	–	–	–	–	–	–
Tigecycline	9	1	1	2	2	–	–	1	1	–	–	1	–	–
Fluoroquinolones^b^	9	2	–	3	1	2	1	–	–	–	–	–	–	–
Colistin	8	1	4	–	1	1	–	–	–	1	–	–	–	–
Ceftriaxone	8	3	–	1	1	1	1	1	–	–	–	–	–	–

^a^Carbapenems refer to meropenem, imipenem, and doripenem

^b^Fluoroquinolones refer to ciprofloxacin and levofloxacin

**Fig. 1:**
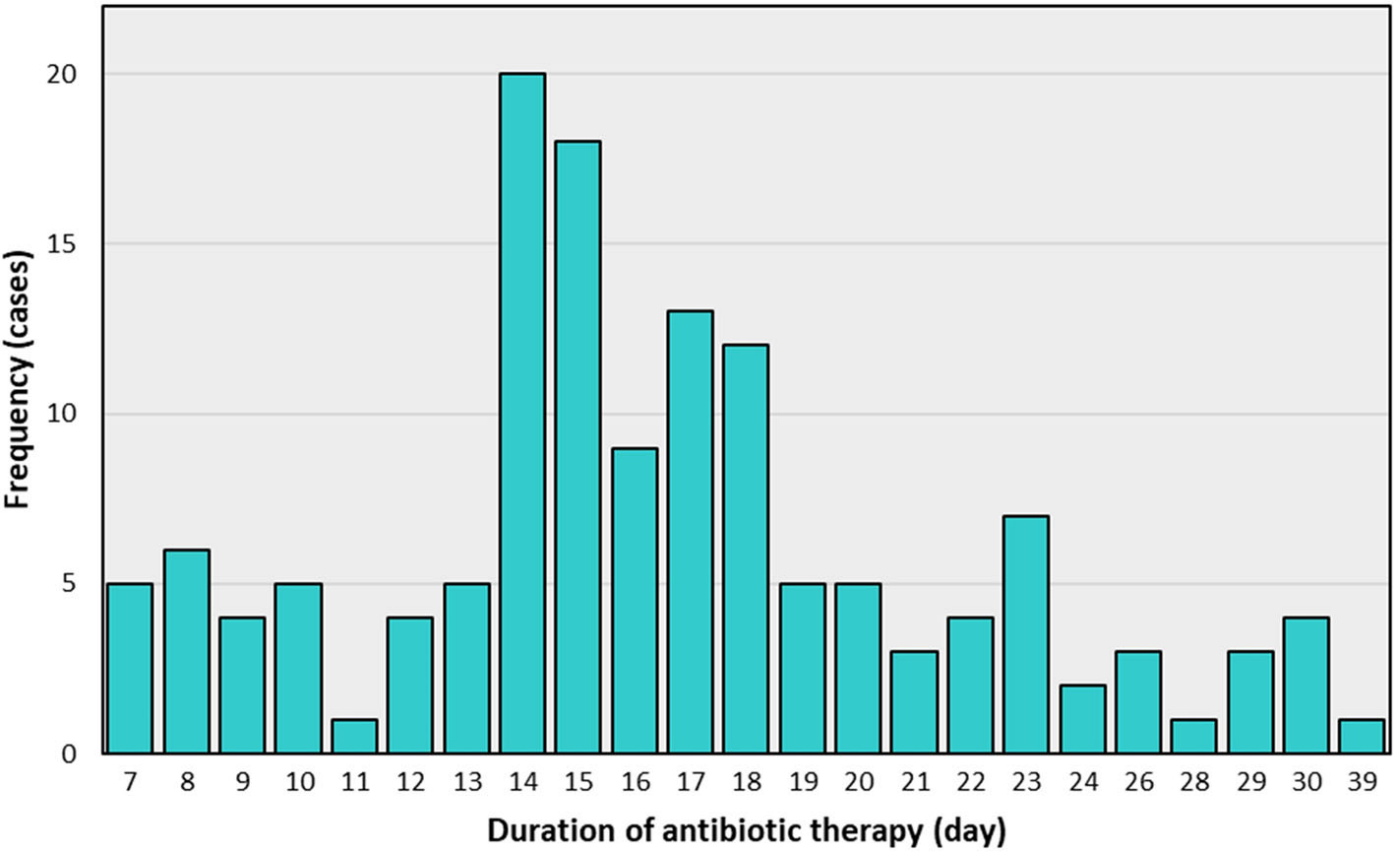
Distribution of antibiotic therapy duration physicians used to treat Gram-negative bacteria catheter-related bloodstream infection

Of the 174 cases of GNB CRBSI, 8 patients did not meet the inclusion criteria (no appropriate antibiotics), whereas 9 patients were excluded according to the exclusion criteria. Another 14 patients were excluded because patients received antibiotics with a duration of less than 7 days, the minimum duration recommended by the IDSA guidelines. One patient was further excluded because the catheter had been removed after completion of antibiotic therapy. In addition, the information on two patients was incomplete because the patients were transferred to another hospital. This left a total of 140 patients for clinical analysis. Patients were prescribed antibiotics for 7 to 39 days. Distribution of the duration of antibiotic therapy is shown in Fig. 1. Fourteen days was chosen as a cut-off point to divide patients into two groups: the long-duration (≥14 day) group and the short-duration (< 14 day) group.

Patients’ characteristics and outcomes in both groups are shown in Table 3. Indications for catheter placement were hemodialysis, nutrition support, medications, hemodynamic monitoring, fluid transfusion, and blood transfusion. We were unable to identify exact indications in some cases, because these data were not routinely recorded in the medical charts. There were only 9 patients with relapse among 140 patients (6.4%). The number of patients and mortality rate were 34 and 24.3%, respectively. Comparing the long-duration group and the short-duration group, overall relapse rates were not different, but greater mortality was observed in the short-duration group. Eleven patients in the short-duration and 8 patients in the long-duration group died during treatment. Thirty-three patients received antibiotics within the 30-day follow-up period (26 patients in the long-duration group and 7 patients in the short-duration group). No difference was found between the two groups in time to next antibiotic use. The patients’ characteristics were compared according to relapse and death. Catheter retention was the only risk factor that was found to significantly increase the risk of relapse (odds ratio = 145.32; 95% confidence interval 12.66–1667.37, *P* < 0.001). No significant risk factor for mortality was found (Table 4).

**Table 3:** Characteristics and clinical outcomes of patients treated with long-duration and short-duration antibiotic in the phase 1 study

Characteristics and outcomes	Long-duration group (*N* = 110)	Short-duration group (*N* = 30)	*p-value*
*Characteristics*			
Age (years)	66 (18–95)	74 (28–97)	0.064
Sex			
Male	59 (53.6%)	19 (63.3%)	0.410
Female	51 (46.4%)	11 (36.7%)	0.410
Charlson comorbidity index (score)	6 (0–14)	6 (0–14)	0.383
Underlying diseases			
HIV infection	1 (0.9%)	0 (0.0%)	1.000
Cancer	27 (24.5%)	12 (40.0%)	0.110
Liver disease	7 (6.4%)	3 (10.0%)	0.446
Renal disease	49 (44.5%)	5 (16.7%)	0.006^a^
COPD	4 (3.6%)	3 (10.0%)	0.169
Diabetes mellitus	42 (38.2%)	7 (23.3%)	0.194
Hematological disease	9 (8.2%)	3 (10.0%)	0.720
Cardiovascular disease	23 (20.9%)	5 (16.7%)	0.798
Trauma	3 (2.7%)	2 (6.7%)	0.291
Immunocompromised status	22 (20.0%)	10 (33.3%)	0.143
Microbial etiology			
Monomicrobial	106 (96.4%)	27 (90.0%)	0.169
Polymicrobial	4 (3.6%)	3 (10.0%)	0.169
Origin of infection			
Healthcare-associated	31 (28.2%)	0 (0.0%)	< 0.001^a^
Nosocomial	79 (71.8%)	30 (100.0%)	< 0.001^a^
ICU admission at onset of infection	49 (44.5%)	22 (73.3%)	0.007^a^
Surgery at present admission	57 (51.8%)	19 (63.3%)	0.305
Type of catheter			
Tunneled hemodialysis catheter	31 (28.2%)	1 (3.3%)	0.003^a^
Short-term central venous catheter or arterial catheter	58 (52.7%)	23 (76.7%)	0.022^a^
Long-term central venous catheter or port	21 (19.1%)	6 (20.0%)	1.000
Catheter management			
Catheter retention	9 (8.2%)	2 (6.7%)	1.000
Catheter removal	101 (91.8%)	28 (93.3%)	1.000
Total duration of therapy (day)	17 (14–39)	10 (7–13)	< 0.001^a^
Duration of therapy after catheter removal (day)	15 (1–28)	8 (3–13)	< 0.001^a^
*Outcome*			
Relapse	7 (6.4%)	2 (6.7%)	1.000
Death^b^	19 (17.3%)	15 (50.0%)	0.001^a^
Time to next antibiotic use (hour)	233.3 (54.0–960.0)	392.5 (102.0–504.0)	0.268
Indication of next antibiotic used			
New infectious episode	13 (11.8%)	2 (6.7%)	0.413
Non-infectious episode	1 (0.9%)	1 (3.3%)	0.384
Relapse	6 (5.5%)	2 (6.7%)	1.000
Undetermined episode	6 (5.5%)	2 (6.7%)	1.000

^a^*p-value* < 0.05

^b^Eleven patients in the short-duration and 8 patients in the long-duration group died during treatment

*Abbreviations: HIV* human immunodeficiency virus, *COPD* chronic obstructive pulmonary disease, *ICU* intensive care unit

Data were presented as frequency (percent) except for age, Charlson comorbidity index, total duration of therapy, duration of therapy after catheter removal, and time to next antibiotic use, which were presented as median (minimum-maximum)

**Table 4:** Risk factors for relapse and death in patients with Gram-negative bacteria catheter-related bloodstream infection

Characteristics	Relapse				Death			
	Yes (*N* = 9)	No (*N* = 131)	*p-value*	Multivariate analysis OR (95% CI), *p-value*	Yes (*N =* 34)	No (*N* = 106)	*p-value*	Multivariate analysis OR (95% CI), *p-value*
Age	69	66	0.171		63	67	0.800	
Sex								
Male	5	73	1.000		22	56	0.241	
Female	4	58			12	50		
Charlson comorbidity index	7	5	0.075		5	6	0.694	
Underlying diseases								
HIV infection	0	1	1.000		0	1	1.000	
Cancer	4	35	0.264		13	26	0.130	
Liver disease	0	10	1.000		4	6	0.256	
Renal disease	5	49	0.307		7	47	0.015^a^	0.630 (0.216–1.895) 0.420
COPD	1	6	0.379		2	5	0.677	
Diabetes mellitus	6	43	0.066		7	42	0.062	
Hematological disease	1	11	0.565		6	6	0.071	
Cardiovascular disease	3	25	0.383		5	23	0.465	
Trauma	0	5	1.000		3	2	0.092	
Immunocompromised status	2	30	1.000		11	21	0.160	
Microbial etiology								
Monomicrobial	9	124	1.000		32	101	0.677	
Polymicrobial	0	7			2	5		
Origin of infection								
Healthcare-associated	3	28	0.415		0	31	< 0.001^a^	266,606,914.2 (< 0.001-infinity) 0.998
Nosocomial	6	103			34	75		
ICU admission at onset of infection	1	70	0.017^a^	0.173 (0.005–6.394), 0.341	26	45	0.001^a^	1.803 (0.590–5.512) 0.301
Surgery at present admission	2	74	0.080		24	52	0.031^a^	0.763 (0.279–2.086) 0.598
Type of catheter								
Tunneled hemodialysis catheter	5	27	0.029^a^	10.023 (0.559–179.714), 0.118	1	31	0.001^a^	1.013 (0.074–13.929) 0.992
Short-term central venous catheter or arterial catheter	2	79	0.036^a^	2.320 (0.072–74.855), 0.635	29	52	< 0.001^a^	2.214 (0.599–8.187) 0.233
Long-term central venous catheter or port	2	25	0.684		4	23	0.317	
Catheter management								
Catheter retention	7	4	< 0.001^a^	145.315 (12.665–1667.369), < 0.001^a^	4	7	0.462	
Catheter removal	2	127			30	99		
Total duration of therapy	14	16	0.269	0.855 (0.623–1.172) 0.330	15	16	0.264	0.990 (0.923–1.062) 0.781
Duration of therapy after catheter removal	13	14	0.946		11	14	0.039^a^	

^a^*p-value* < 0.05

*Abbreviations: HIV* human immunodeficiency virus, *COPD* chronic obstructive pulmonary disease, *ICU* intensive care unit

Data were presented as count except age, Charlson comorbidity index, total duration of therapy, duration of therapy after catheter removal, which were presented as median

In the prospective phase 2 study, there were 80 patients with GNB CRBSI. However, inclusion criteria were met for only 27 patients. Sixteen patients were excluded according to exclusion criteria (4 with catheter retention, which was the only risk factor for relapse found in the phase 1 study; 1 with ongoing neutropenia; 1 with abdominal abscess; 1 with osteomyelitis; 1 with septic arthritis; 4 with other indications for antibiotic continuation; and 1 due to patient’s refusal to participate). One case was withdrawn because the patient was transferred to another hospital and 2 cases were withdrawn due to death before antibiotic discontinuation, leaving 11 patients recruited into the short-duration group. The patients in this group were mainly immunocompetent, with nosocomial infections. The catheter type and indication varied. During the follow-up period, no relapse was found. Antibiotics were restarted in 3 patients for conditions unrelated to the GNB CRBSI. Surveillance hemocultures were performed 7 days after antibiotic discontinuation except for 2 patients, from whom hemocultures were taken on day 8 due to outpatient hospital visits. The results were negative except in one patient, whose culture was positive for *K. pneumoniae* and *Candida glabrata*, with the diagnosis of intra-abdominal infection with septicemia.

Thirty-three matched patients with catheter removal from the phase 1 study who received antibiotics ≥14 days were selected for the long-duration group. Comparisons of patients’ characteristics and clinical outcomes between the long- and short-duration groups are shown in Table 5. Outcomes were comparable between the two groups.

**Table 5:** Comparison of patients with catheter removal receiving long- and short-duration antibiotic in the phase 2 study

Characteristics and outcomes	Long-duration group (*N* = 33)	Short-duration group (*N =* 11)	*p-value*
*Characteristics*			
Age (year)	67 (18–81)	68 (24–81)	0.979
Gender			
Male	16 (48.5%)	5 (45.5%)	1.000
Female	17 (51.5%)	6 (54.5%)	1.000
Charlson comorbidity index (score)	5 (0–12)	4 (0–10)	0.936
Underlying diseases			
HIV infection	0 (0.0%)	0 (0.0%)	–
Cancer	7 (21.2%)	3 (27.3%)	0.692
Liver disease	1 (3.0%)	1 (9.1%)	0.442
Renal disease	16 (48.5%)	4 (36.4%)	0.728
COPD	1 (3.0%)	0 (0.0%)	1.000
Diabetes mellitus	13 (39.4%)	5 (45.5%)	0.738
Hematological disease	2 (6.1%)	1 (9.1%)	1.000
Cardiovascular disease	8 (24.2%)	7 (63.6%)	0.028^a^
Trauma	1 (3.0%)	0 (0.0%)	1.000
Immunocompromised status	6 (18.2%)	2 (18.2%)	1.000
Microbial etiology			
Monomicrobial	31 (93.9%)	10 (90.9%)	1.000
Polymicrobial	2 (6.1%)	1 (9.1%)	1.000
Origin of infection			
Healthcare-associated	9 (27.3%)	1 (9.1%)	0.408
Nosocomial	24 (72.7%)	10 (90.9%)	0.408
ICU admission at onset of infection	13 (39.4%)	4 (36.4%)	1.000
Surgery at present admission	18 (54.5%)	6 (54.5%)	1.000
Type of catheter			
Tunneled hemodialysis catheter	10 (30.3%)	2 (18.2%)	0.698
Short-term central venous catheter or arterial catheter	19 (57.6%)	4 (36.4%)	0.303
Long-term central venous catheter or port	4 (12.1%)	5 (45.5%)	0.030^a^
Total duration of therapy (day)	17 (14–30)	7 (7–10)	< 0.001^a^
Duration of therapy after catheter removal (day)	14 (1–23)	7 (7–7)	< 0.001^a^
*Outcome*			
Relapse	1 (3.0%)	0 (0.0%)	1.000
Death	4 (12.1%)	1 (9.1%)	1.000
Time to next antibiotic use (hour)	357.25 (54.0–444.0)	224.0 (176.0–312.75)	0.400
Indication of next antibiotic used			
New infectious episode	3 (9.1%)	2 (18.2%)	1.000
Non-infectious episode	0 (0.0%)	0 (0.0%)	–
Relapse	1 (3.0%)	0 (0.0%)	1.000
Undetermined episode	0 (0.0%)	1 (9.1%)	0.429

^a^*p-value* < 0.05

*Abbreviations: HIV* human immunodeficiency virus, *COPD* chronic obstructive pulmonary disease, *ICU* intensive care unit

Data were presented as frequency (percent) except for age, Charlson comorbidity index, total duration of therapy, duration of therapy after catheter removal and time to next antibiotic use, which were presented as median (minimum-maximum)

## Discussion

During the 7-year duration of the phase 1 study, there were 174 cases of patients with GNB CRBSI. This low number of cases was due to the strict IDSA diagnostic criteria used, which required laboratory evidence to confirm that the catheter was the source of infection [8], which is different from many studies that use central line-associated bloodstream infection (CLABSI) definition. CLABSI is defined as a bloodstream infection in a patient who had a central line within the 48-h period before the infection occurs. CLABSI may overestimate the true incidence of CRBSI and is used for surveillance purpose [11]. The most common pathogens found in our study were *P. aeruginosa.* These findings are similar to studies in Israel and Spain that reported *P. aeruginosa* as the CRBSI pathogen for approximately 28% of cases [5, 7]. Approximately half of cases in our study were breakthrough infections while on antibiotic therapy. Carbapenems were the most common antibiotics used at that time. The most common pathogens causing breakthrough infections were *Acinetobacter* spp., *Pseudomonas* spp., and *S. maltophilia*., all of which are major problems in terms of drug-resistant, nosocomial, GNB pathogens. To the best of our knowledge, this is the first report of microbial epidemiology in breakthrough GNB CRBSI.

From the 140 patients included in clinical analysis, most patients had been prescribed with long-duration therapy, given using a 14-day cut-off resulted in a small number of patients with short-duration therapy. Long-duration antibiotics were more likely prescribed in patients with underlying renal disease, healthcare-associated infections, or tunnel hemodialysis catheter. However, none of these factors were reported to be risk factors for relapse or death. This showed that physicians here still preferred to extend the duration of treatment even though the IDSA guidelines suggested a minimum duration of 7 days [8]. The possible explanation was short duration of antibiotic treatment was not a common practice in our setting. Moreover, as there is universal health-care coverage scheme in Thailand, there was no need for the patients to pay for the cost of long-duration antibiotic treatment. Other guidelines from the Spanish Society of Infectious Diseases and Clinical Microbiology and the Spanish Society of Spanish Society of Intensive and Critical Care Medicine and Coronary Units have suggested that the duration of therapy should be individualized, and at least 7-day antibiotic treatment has been recommended [12].

Compared with reported relapse rates between 7.5 and 11.1%, a low relapse rate of only 6.4% was found in our phase 1 study [13–15]. There was no difference in relapse rates between patients in the long- and short-duration groups. From the bivariate analysis, despite three factors that could affect the outcome (ICU admission at onset of CRBSI, type of catheter, and catheter management), only catheter retention was strongly associated with relapse. This is consistent with a previous study of children with uncomplicated GNB bacteremia in which the main sources of infection were CRBSI, with reported catheter retention and polymicrobial bacteremia as risk factors for relapse [16]. On the other hand, catheter removal had also been reported as an independent protective factor against relapse in a study of GNB CRBSI [15]. Relapse in patients without catheter removal could be related to biofilm formation on the external surface or inner lumen of the catheter [17]. In addition to Gram-positive bacteria, GNB were able to produce biofilm, as shown in an in vitro system in which human blood components had promoted adhesion and biofilm formation [18]. Apart from catheter removal, prolonged-duration of antibiotics does not show evidence to support reduced relapse risk [14, 16].

For the fatal outcome, the short-duration group in the phase 1 study had significantly more deaths than the long-duration group. This could be explained by the fact that patients who died during antibiotic treatment were not excluded. From the 30 patients in the short-duration group, 11 patients (36.7%) were recruited into this group because antibiotics were discontinued due to their deaths, whereas only 8 patients (7.3%) died before antibiotic discontinuation among the 110 patients in the long-duration group. In addition, we could not find that antibiotic duration was associated with death at all. Characteristics of patients in the short-duration group were more likely to be patients with nosocomial infection, ICU admission at onset of infection, and short-term central venous catheter. This might reflect the patients’ severity of illness that could have resulted in more deaths. Despite the many possible risk factors for death that were found in the bivariate analysis (underlying renal disease, origin of infection, ICU admission at onset of CRBSI, surgery at present admission, duration of antibiotic after catheter removal, and type of catheter), no significant risk factor was found in the multivariate analysis. Compared with previously reported risk factors for death, which were catheter retention [19], surgery in the present admission, infection caused by *P. aeruginosa*, complicated bacteremia (recurrence, septic arthritis, wound infection) [13], male patients, and catheterizations with triple lumen type catheters [20], our study found that surgery at present admission and type of catheter (short-term central venous catheter or arterial catheter and tunneled hemodialysis catheter) were also possible risks but were not found to be significant in the multivariate analysis. Catheter retention and male patients were not found to be significant risk factors in this study. Infection caused by *P. aeruginosa* was not tested due to various other pathogens found. Complicated bacteremia was excluded from the exclusion criteria.

In the 1-year-prospective part of the study (phase 2), there were only 11 patients recruited to receive short-duration antibiotic treatment. Reasons for the low number of participants were due to the strict diagnostic criteria used, and because only patients with catheter removal were selected. After matching with patients receiving long-duration therapy, patients in the short-duration group had significantly more underlying cardiovascular diseases and types of catheterization: long-term central venous catheter or port. However, these characteristics showed no association with focused clinical outcome as no significant risk factors for relapse or death were found from a bivariate analysis (data not shown). Comparing these matched patients, outcomes between the 2 groups were not different. Despite the short-duration of therapy, no relapse was found. To the best of our knowledge, this study is the first prospective study to evaluate the outcome of short-duration treatment of uncomplicated GNB CRBSI in patients with catheter removal. Results show that prolonged-duration of antibiotics might not have additional benefits in the prevention of relapse, because relapse risk should already be low after the catheters have been removed. This supports the concept of shortening the duration of antibiotic therapy, evidence for which is increasing for many infectious diseases [21–23]. A study in children with uncomplicated GNB bacteremia, in which CRBSIs were the main sources of infection, found that prolonged antibiotic treatment did not reduce relapse risk [16]. Another study of patients with GNB CLABSI in the surgical trauma ICU found similar duration of therapy in patients with and without recurrence [14]. Nevertheless, these two studies included patients without catheter removal. Other studies of patients with GNB bacteremia from various source of infection, including CRBSI, found no difference in many outcomes, which were relapse, death, *Clostridium difficile* infection, suppurative or distant complications, readmission, or extended hospitalization between patients treated with long- and short-duration therapy [24–26].

This study collected data on many cases of GNB CRBSI over a long period of time. Our strict diagnostic criteria could be a strength of our study, given the criteria might specify catheters as the true primary source of bloodstream infection. Various pathogens, including multidrug-resistant nonfermented GNB, have been reported. Unlike most of the other studies that have been conducted in patients with uncomplicated GNB bacteremia from *Enterobacteriaceae* as the main pathogens, results from this study can be specifically applied for patients with CRBSI. One limitation in this study was that patients who died before completing the course of treatment were included, thus, the duration of therapy among these patients did not reflect the true duration. However, all patients had already received antibiotics for at least 7 days, which was the suggested minimum duration, and short duration of antibiotics was not found to increase risk of death. Only a few patients could be enrolled in the prospective study due to the limitation of a 1-year study period and the hospital’s infection control policy of the CLABSI bundle to reduce risk of CRBSI leading to lower prevalence of this condition. However, results from this study could lead to larger trials on the efficacy of shortening the duration of antibiotic therapy for uncomplicated GNB CRBSI.

## Conclusions

We confirm that catheter removal is crucial for the management of GNB CRBSI, because that is the most important action that can be performed to prevent relapse. The empiric antibiotic should be broad-spectrum, with coverage of *P. aeruginosa.* In addition, short-duration treatment is possible for patients with uncomplicated GNB CRBSI who have had their catheters removed.

## Data Availability

The datasets used and/or analyzed during the current study are available from the corresponding author.

## Abbreviations

CRBSI: Catheter-related bloodstream infection
GNB: Gram-negative bacteria
IDSA: The Infectious Diseases Society of America
HIV: Human immunodeficiency virus
COPD: Chronic obstructive pulmonary disease
ICU: Intensive care unit
CLABSI: Central line-associated bloodstream infection
